# WE-ASCA: The Weighted-Effect ASCA for Analyzing Unbalanced Multifactorial Designs—A Raman Spectra-Based Example

**DOI:** 10.3390/molecules26010066

**Published:** 2020-12-25

**Authors:** Nairveen Ali, Jeroen Jansen, André van den Doel, Gerjen Herman Tinnevelt, Thomas Bocklitz

**Affiliations:** 1 Institute of Physical Chemistry and Abbe Center of Photonics (IPC), Friedrich-Schiller-University, Helmholtzweg 4, D-07743 Jena, Germany; nairveen.ali@uni-jena.de; 2 Leibniz Institute of Photonic Technology (Leibniz-IPHT), Member of Leibniz Research Alliance Health Technologies, Albert-Einstein-Strasse 9, D-07745 Jena, Germany; 3 Institute for Molecules and Materials (IMM), Radboud University, Heyendaalseweg 135, 6525 AJ Nijmegen, The Netherlands; jj.jansen@science.ru.nl (J.J.); h.vandendoel@science.ru.nl (A.v.d.D.); G.Tinnevelt@science.ru.nl (G.H.T.); 4 TI-COAST, Science Park 904, 1098 XH Amsterdam, The Netherlands

**Keywords:** ASCA, unbalanced experimental design, general linear model, weighted-effect coding, biomedical Raman spectra

## Abstract

Analyses of multifactorial experimental designs are used as an explorative technique describing hypothesized multifactorial effects based on their variation. The procedure of analyzing multifactorial designs is well established for univariate data, and it is known as analysis of variance (ANOVA) tests, whereas only a few methods have been developed for multivariate data. In this work, we present the weighted-effect ASCA, named WE-ASCA, as an enhanced version of ANOVA-simultaneous component analysis (ASCA) to deal with multivariate data in unbalanced multifactorial designs. The core of our work is to use general linear models (GLMs) in decomposing the response matrix into a design matrix and a parameter matrix, while the main improvement in WE-ASCA is to implement the weighted-effect (WE) coding in the design matrix. This WE-coding introduces a unique solution to solve GLMs and satisfies a constrain in which the sum of all level effects of a categorical variable equal to zero. To assess the WE-ASCA performance, two applications were demonstrated using a biomedical Raman spectral data set consisting of mice colorectal tissue. The results revealed that WE-ASCA is ideally suitable for analyzing unbalanced designs. Furthermore, if WE-ASCA is applied as a preprocessing tool, the classification performance and its reproducibility can significantly improve.

## 1. Introduction

An essential part of statistical analysis is the extraction of informative features that describe a specific phenomenon based on a limited number of samples. These samples are mostly collected by conducting either experiments or surveys [1,2]. In survey studies, a large number of individuals are involved to collect information without changing the existing conditions of the studied phenomenon. The other type of sampling is to conduct an experiment that tests the effect of one, or more than one, treatment on selected individuals. This experimental approach is widely applied in the fields of the physical and life sciences. In such studies, an experiment is carefully designed so that the obtained results are objective and valid [3,4]. Here, the term “design of experiment” (DoE) refers to statistical techniques that deal with planning and analyzing controlled tests, which investigate the effect of the studied treatments on selected individuals. There are several techniques for designing the experiments and one of the most common designs is the factorial design [5]. Therein, experiments are planned to extract information based on investigating the effect of at least two treatments in one experiment [6]. These treatments are termed the experimental factors, and the combinations of these factors define their interactions.

Within biomedical studies, e.g., testing the efficiency of a new drug or checking a new technique for disease detection, the effect of experimental factors and their interactions are translated into different types of variations that can be categorized into two main groups: informative (or interesting) variations and disturbing (or unwanted) variations. The informative variations highlight the differences between different states like sample properties or disease states. In contrast, disturbing variations may be assigned to systematic perturbations within the experiment, which might negatively affect the results of further analyses. The later variation is very difficult to be controlled, and it mostly arises when many devices or different individuals are considered to perform an experiment. In this discourse, multifactorial analysis methods were introduced as powerful techniques to understand and analyze the variations within the factorial experimental design. The basic idea here is built upon hypothesis testing of more than two groups referring to factor levels. These factorial analysis tests were established quite well for univariate data, and they are known as analysis of variance (ANOVA) tests [1,7,8]. In one of their classical versions, namely the one-way ANOVA test, the effect of one factor on selected observations is studied based on testing the mean differences between the factor levels. The multi-way ANOVA tests search in a multifactorial design for significant effects based on checking the differences between the levels of each factor and each factor interaction. However, if the response data set is described by multiple features, only a few methods of multifactorial design were developed, which typically feature some limitations. For instance, in the basic form of multivariate-ANOVA (MANOVA) tests, an ANOVA test is performed for several response variables, which allows for studying the effect of one or more than one factor on these response variables [9,10]. The main restriction here is that performing MANOVA tests require a large number of measurements, e.g., sample size, compared to the number of variables (features). Such data are often not available, especially in modern technologies which introduce high dimensional measurements like spectra or images. Therefore, combining principal component analysis (PCA) models with ANOVA tests provided a solution to deal with the high dimensionality of response matrices in multifactorial designs [11]. The PC-ANOVA starts by fitting the response matrix with a PCA model, then the obtained principal components (PCs) are analyzed using ANOVA tests. Although the PC-ANOVA does not have the limitation respecting the sample size and number of response variables, some information related to factor contributions might be missed during the PCA projection. Besides, ANOVA simultaneous component analysis (ASCA) was presented as a powerful tool to deal with multivariate data in multifactorial designs [12,13]. In brief, ASCA methods decompose the response matrix into different effect matrices, which characterize the contribution of each effect in the designed model. These contributions are measured by the amount of variance explained by each effect. Thereafter, ASCA checks which effect contributes significantly to the considered model, and finally, the dimensions of each effect matrix are reduced based on a PCA model or a SCA model [14,15]. Later improvements of ASCA were introduced based on scaling the response matrix first, then applying the classical ASCA pipeline [16]. In this reference, it was shown how the considered scaling approach can affect the interpretation of the ASCA results. However, the proposed design of ASCA and its improvements are valid only for balanced designs, in which the levels of each factor have equal numbers of measurements. This constraint creates an additional limitation to the application area of ASCA. Thus, the ASCA+ was introduced later as an extension of ASCA to deal with unbalanced designs [17]. It utilizes general linear models (GLMs) to decompose the response matrix into two main terms: The estimated response matrix and the residual matrix, which refers to the estimation error [8,17]. Within ASCA+, the levels of each effect are coded using the deviation coding that has a main advantage concerning the variance maximization produced if the classical ASCA is applied on unbalanced designs [18].

In the paper, we review the implementation of ASCA and ASCA+ in unbalanced designs, and we introduce an updated extension of ASCA based on weighted-effect (WE) coding as a powerful tool to deal with these unbalanced designs. This WE-coding is a type of dummy coding that offers an attractive feature in which the sum of all level effects of a categorical variable is equal to zero [19,20]. Additionally, the results of WE-coding are identical to those obtained by deviation coding in balanced designs. The new ASCA extension, named WE-ASCA, substitutes the deviation coding of the design matrix in the ASCA+ method by the WE-coding in order to estimate the contributions of experiment effects. The performance of WE-ASCA was evaluated based on a Raman spectral data set of 47 individual mice for two different applications. In the first task, we analyzed the complex multifactorial design presented within the Raman data set using WE-ASCA, and we compared its results with the obtained ones by ASCA and ASCA+. Thereafter, the WE-ASCA was implemented as a preprocessing technique to improve the tissue classification. This was accomplished by applying the WE-ASCA to exclude disturbing variations from the training set. Later, a classification model was constructed using the new training set only, i.e., without disturbing variations, while the classification results based on WE-ASCA were compared with those obtained by the same classifier trained without using WE-ASCA-based preprocessing.

## 2. Results

**Figure 1:**
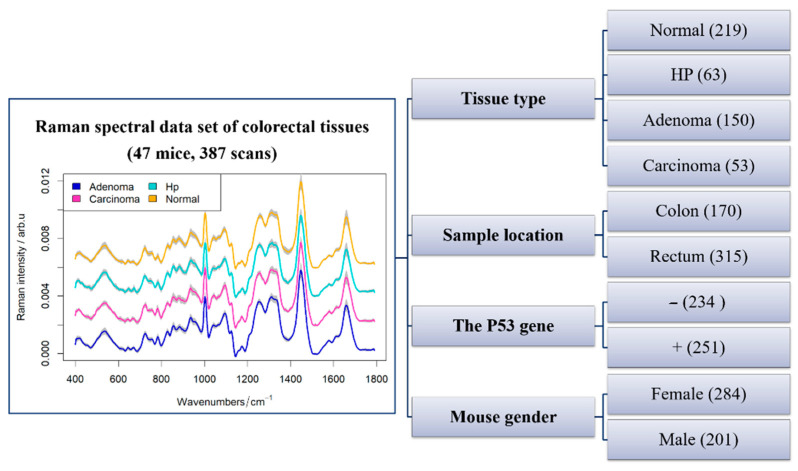
An overview of the experimental design of the Raman spectral data set. The studied data set consists of 47 mice and 387 scans that were collected from four different tissue types. The means spectra of these tissue types are shown in the left side. In this experiment, three factors were investigated: the activity of the P53 gene (active and inactive), the mouse gender, and the sample location (colon and rectum). It is observed that the number of scans of factor levels is different.

Two applications of WE-ASCA are demonstrated in this section based on an unbalanced multifactorial design of a Raman spectral data set comprising 387 colorectal tissue scans that were collected from 47 mice. Within this study, the activity of the P53 gene (active +, inactive -) and the mice gender (male, female) were recoded while the Raman spectra of each scan were annotated as different tissue “types” representing normal, hyperplasia (HP), adenoma, and carcinoma tissue. Later, the biological variations of these colorectal tissues extracted from mice rectum or colon were evaluated based on the acquired Raman spectral scans [21]. In the presented work, a mean spectrum per tissue type was calculated resulting in 485 Raman spectra acquired from 387 scans. The number of mean spectra and number of scans differ because in one scan multiple tissue types can be present, yielding a higher number of mean spectra per scan. For example, a scan may contain cancer tissue and normal tissue, leading to two mean spectra for this scan. Figure 1 depicts the mean spectra of the tissue types beside the design of our experiment. Therein, the factors exhibit the sample location, the mouse gender, and the activity of the P53 gene. Notably, the number of spectra within the levels of each factor is different; thus, the introduced WE-ASCA is ideal for analyzing this unbalanced design.

In the following, the contributions of experimental factors in addition to their interactions are estimated based on the classical ASCA and its extensions ASCA+ and WE-ASCA. Then, an evaluation for the variance explained by each effect was performed. In the second subsection, the WE-ASCA is applied as a preprocessing technique to assess its performance in improving the classification of colorectal tissues.

### 2.1. A Comparison between Analyses of Multifactorial Design Using ASCA, ASCA+ and WE-ASCA

Since 47 mice were included to perform this study, an additional variation connected to the biological differences between mice might be produced. We added therefore another factor featuring the individuals (mice) contribution to the experimental design. Consequently, the multifactorial model that describes the considered experiment was built upon the individual factor with 47 levels indicating the mice, the activity of the P53 gene, the mouse gender, and the sample location (colon or rectum) in addition to interactions between the last three factors. This mathematical model can be formulated as:

(1) $$X=M_{0}+{\mathrm{Con}}_{\mathrm{Individual}}+{\mathrm{Con}}_{\mathrm{Location}}+{\mathrm{Con}}_{P53}+{\mathrm{Con}}_{\mathrm{Gender}}\;\;\;\;\;\;+{\mathrm{Con}}_{\mathrm{Loction}:P53}+{\mathrm{Con}}_{\mathrm{Location}:\mathrm{Gender}}\;+{\mathrm{Con}}_{P53:\mathrm{Gender}}+\hat{E},$$

where $X\in {\mathbb{R}}^{485\times 696}$ is the Raman spectral matrix, $M_{0}$ denotes a matrix in which mean Raman spectra with respect to the wavenumbers are oriented in rows, and $Con_{f}$ represents the matrix of an effect f. Based on this model, we evaluated the results of classical ASCA and its extensions ASCA+ and WE-ASCA using the obtained percentages of variances. As it is displayed in Table 1, the residual matrix of all analyses introduced the largest percentage of variance while the individual factor produced the largest factor contribution among all other effects. Here, the percentages of variance explained by the individuals are 37.97%, 33%, and 33.94% when ASCA, ASCA+, and WE-ASCA are applied, respectively. In contrast, the remaining factors and their interactions showed quite small contributions to the overall variance if any of the three multifactorial analyses were implemented. Nevertheless, the sum of percentages of variances by ASCA, ASCA+, and WE-ASCA is 108.62%, 93.3%, and 96.01%, respectively. This means that the classical ASCA maximized the effect contributions while the ASCA+ analysis minimized these contributions; however, the WE-ASCA analysis introduced the best estimations of effect contributions among the other analyses.

**Figure 2:**
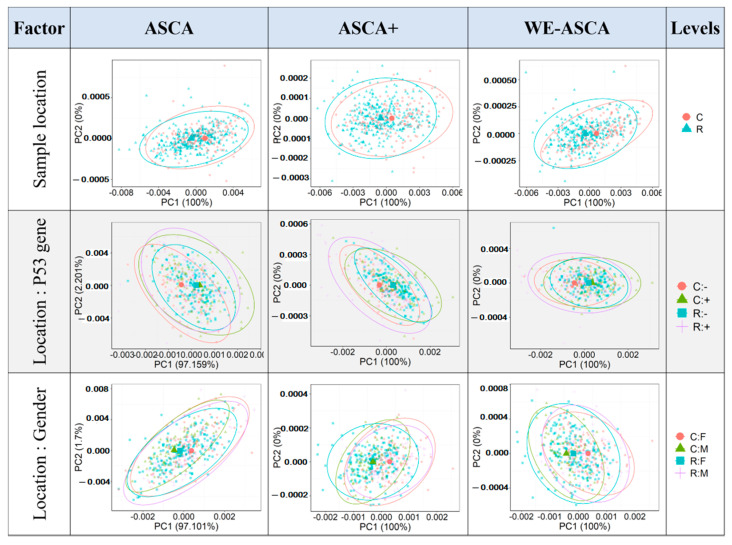
The score plots of first two principal components (PCs) of principal component analysis (PCA) sub-models using ASCA, ASCA+ and WE-ASCA. The WE-ASCA analysis provides better separation between the group means in comparison to the results obtained by the classical ASCA or its extension ASCA+.

The obtained results in Table 1 show that only the individual factor and the sample location in addition to its interactions with the P53 gene and the mouse gender contributed to explain the variance in the considered design. To interpret the inner variance of these effects, a sperate PCA model was fitted to each of previous effect matrices, then the obtained PCA results were summarized in Table 2 and Figure 2. The column named “all data” in Table 2 shows the percentages of variance explained by the first two PCs if the spectral matrix X was mean centered and projected by a PCA model. These two PCs explained around 57.72% of the overall variance. For the PCA sub-model of the individual effect and the sample location, the three multifactorial analyses estimated approximately the same percentage of variance by the first two PCs. Therein, the first two PCs of the individual sub-models explained between 53.68% and 55.57% of the variance presented in the individual effect matrix, while the first PC estimated almost the whole variance of the sample location effect. Moving to the interaction effects, the first PC could describe almost 100% of the effect’s variance if ASCA+ and WE-ASCA were applied. But this variance estimation was different in the case of classical ASCA, where the first PC explained around 3% variance less of this interaction effect. In Figure 2, the score plots of the PCA sub-models extracted from the effect matrix of the sample location and its interaction with the P53 gene and mouse gender are presented. The points in this figure depict the sum of the contribution of each effect and the projection of the residual matrix on the loadings obtained by the PCA sub-models (see [22] for more details). The bold points represent the group means and the ellipses are a representation of the covariance matrix. The WE-ASCA here shows slightly better separation between the group means in comparison to the results obtained by the ASCA and ASCA+.

After calculating the effect contributions and the PCA sub-models, we determined which effects contributed significantly to the experimental design using both ASCA extensions, i.e., ASCA+ and WE-ASCA, based on the described permutation test with N=1000 iterations. The obtained p-values p(f) by these tests were combined and presented in Table 3. Whether ASCA+ or WE-ASCA are applied, the individual factor and the sample location factor caused a significant effect in the design of our experiment. Here, the obtained p(f) of the individual factor is almost zero by both analyses, while the p(f) of the sample location is 0.021% and 0.034% when WE-ASCA and ASCA+ are applied, respectively.

To conclude, the WE-ASCA improved the analysis of unbalanced multifactorial design within the considered Raman data set. This improvement was detected by comparing the sum of explained variance obtained by the classical ASCA and its extension ASCA+ with the results of the presented WE-ASCA. The analysis of this experiment yielded that the effect of the individual factor produced the highest variation and the most significant effect within the studied data set. 

### 2.2. A WE-ASCA as Preprocessing Technique in Classification Models

**Figure 3:**
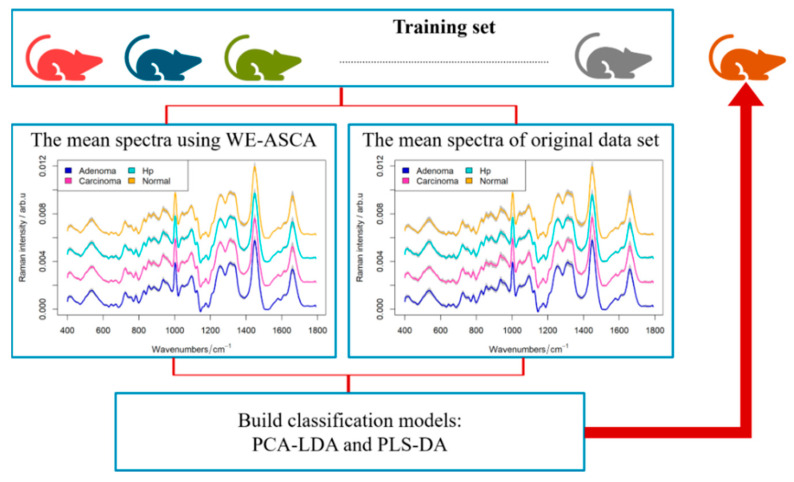
Overview of classification pipelines using WE-ASCA as a preprocessing step. Herein, a leave-one-mouse-out cross-validation (LOMO-CV) is implemented, while WE-ASCA is performed for each iteration within the CV loop. We can see that the variation of mean spectra after applying the WE-ASCA is smaller than the variation of mean spectra of the tissue types without preprocessing the spectral data. Based on training sets with or without applying WE-ASCA as preprocessing step, a PCA-LDA and PLS-DA are constructed, and their performance was determined.

The goal of this subsection is to assess the performance of the ASCA analyses as a preprocessing technique. Therein, the WE-ASCA was implemented within the cross-validation as a preprocessing tool in order to exclude disturbing variations. In our work, a leave-one-mouse-out cross-validation was performed to check the results of two classifiers, namely the combination of a principal component analysis with a linear discriminant analysis (PCA-LDA) and the combination of a partial least square regression with a linear discriminant analysis (PLS-DA). The classification and validation procedure starts by fixing spectra of a specific mouse $T_{i}:i=1,\;2,\;\ldots ,\;47$ as a test set and training the classifiers using the Raman spectra of the remaining mice, e.g., $X\left(-T_{i}\right)$. This procedure is iterated until spectra of all mice are predicted once while the WE-ASCA is always applied on the training set of each cross-validation iteration (see Figure 3). It was shown in Section 2.1 that the individual factor produced the highest percentage of variance, which might negatively affect the classification results. Therefore, a new training set $X_{\mathrm{ASCA}}\left(-T_{i}\right)$ is estimated by excluding the variation of these individuals, then the training set $X_{\mathrm{ASCA}}\left(-T_{i}\right)$ can be defined as:

(2) $$X_{\mathrm{ASCA}}\left(-T_{i}\right)=X\left(-T_{i}\right)-{\mathrm{Con}}_{\mathrm{Individual}}\left(-T_{i}\right);\left(i=1,\;2,\;\ldots ,\;47\right),$$

where $Con_{Individual}\left(-T_{i}\right)$ denotes to the individual effect matrix obtained by applying the WE-ASCA on $X\left(-T_{i}\right)$. Using $X_{\mathrm{ASCA}}\left(-T_{i}\right)$ and $X\left(-T_{i}\right)$, the PCA-LDA model and PLS-DA model are constructed. Then the tissue labels of each scan are predicted by both classifiers.

**Figure 4:**
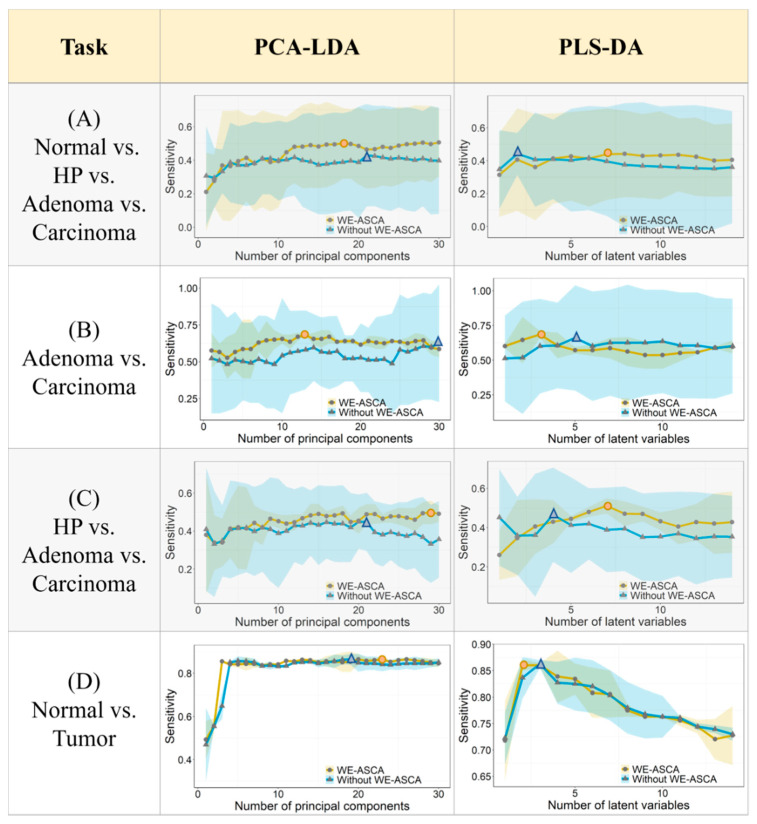
A comparison between the classification results of principal component analysis with a linear discriminant analysis (PCA-LDA) and partial least square regression with a linear discriminant analysis (PLS-DA) models based on leave-one-mouse-out cross-validation. Each classifier was trained twice with and without applying WE-ASCA-based preprocessing. The blue lines and the blue regions show the mean sensitivity and the standard deviation of a classifier constructed on the spectra without applying WE-ASCA on the training set. The yellow lines and the yellow regions depict the mean sensitivity and the standard deviation of a classification model trained on training sets that were preprocessed using WE-ASCA. The elimination of individual variations based on WE-ASCA improved the classification performance, and it significantly reduced the variance within the cross-validation results: (**A**) The maximum mean sensitivity for the differentiation between the scans of normal, HP, adenoma, and carcinoma tissues is 50.67%, and it was reached when training a PCA-LDA model on spectra processed by WE-ASCA. (**B**) For the classification of adenoma and carcinoma tissues, the maximum mean sensitivity of PCA-LDA (PLS-DA) is 67.98% (68.47%). These results were also achieved if the training sets were processed based on the WE-ASCA. (**C**) WE-ASCA-based preprocessing improved the differentiation between the three suspicious tissues. The maximum mean sensitivity of PCA-LDA model and PLS-DA are 49. 85% and 51.09%, respectively. (**D**) The results of differentiating the normal and tumor tissue. While, training an PCA-LDA model with or without spectra processed by WE-ASCA provided almost the same classification results, training a PLS-DA model on spectra processed by WE-ASCA improved the mean sensitivity and decreased thestandard deviation.

The previous procedure was tested on four classification tasks, and the results are presented in Figure 4. The columns of this figure describe the results of PCA-LDA and PLS-DA models while the rows show the cross-validation results of each task for a different number of PCs or latent variables. If an LDA was trained on $X_{\mathrm{ASCA}}\left(-T_{i}\right)$, the standard deviation and the mean sensitivity were presented by the yellow regions and the yellow lines. In the other case, the blue regions and blue lines indicate the standard deviation and the mean sensitivity of LDA models constructed by $X\left(-T_{i}\right)$ without preprocessing by WE-ASCA. In Figure 4A, the classification means sensitivities of PCA-LDA and PLS-DA are presented in order to differentiate between the scans of normal, HP, adenoma, and carcinoma tissues. It is observed that both classifiers produced better results when they were trained by the $X_{\mathrm{ASCA}}\left(-T_{i}\right)$. Here, the maximum mean sensitivity of the PCA-LDA model trained on $X_{\mathrm{ASCA}}\left(-T_{i}\right)$ is 50.67%. If the same classifier was built on the training set $X\left(-T_{i}\right)$, the mean sensitivity decreased to 42.93%. Constructing a PLS-DA model on a $X_{\mathrm{ASCA}}\left(-T_{i}\right)$ or on $X\left(-T_{i}\right)$ presented almost the same mean sensitivity, but it showed narrower standard deviation regions. For classifying adenoma and carcinoma tissues, it is clear in Figure 4B that using the WE-ASCA in preprocessing Raman spectra improved the LDA performance. While the maximum mean sensitivity of PCA-LDA and PLS-DA without implementing the WE-ASCA was 62.56% and 66%, respectively, the maximum mean sensitivity of PCA-LDA and PLS-LDA based on the training sets $X_{\mathrm{ASCA}}\left(-T_{i}\right)$ increased to 67.98% and 68.47%, respectively. Moving to Figure 4C, which represents the classification results of the three suspicious tissues, WE-ASCA significantly improved the classification results, which can be observed as an increase in the mean sensitivity and a decrease of the standard deviation of PCA-LDA and PLS-DA models if they are trained by $X_{\mathrm{ASCA}}\left(-T_{i}\right)$. In this case, the maximum mean sensitivity of PCA-LDA and PLS-LDA models increased at least 5% if they are constructed on the training sets $X_{\mathrm{ASCA}}\left(-T_{i}\right)$. The last classification task aimed to differentiate between normal tissues and tumor tissues combining carcinoma and adenoma spectra in one class. The obtained results are presented in Figure 4D. Both classifiers provided almost the same maximum mean sensitivity based on the training sets $X_{\mathrm{ASCA}}\left(-T_{i}\right)$ and $X\left(-T_{i}\right)$. However, training the classification models on $X_{\mathrm{ASCA}}\left(-T_{i}\right)$ decreased the standard deviation and required less latent variables for constructing PLS-LDA models. Nonetheless, utilizing WE-ASCA in preprocessing Raman spectra enhanced the results reproducibility of classification models. This reproducibility improvement is clearly seen in Figure 4 as narrower variation regions when the LDA models were trained by the $X_{\mathrm{ASCA}}\left(-T_{i}\right)$. Here, the standard deviations of both classifiers built on $X_{\mathrm{ASCA}}\left(-T_{i}\right)$ decreased significantly compared to the standard deviations of the same models trained by $X\left(-T_{i}\right)$.

Overall, the WE-ASCA-based spectra al preprocessing allowed us to exclude the disturbing variation from the training data set, and it significantly improved the classification performance. This improvement can be detected as an increase in the classification mean sensitivities and a reduction of the variance in the cross-validation results. Furthermore, the WE-ASCA-based spectral preprocessing required a smaller number of principal components (or latent variables) to build the classification models since the data distortion in the training data set was eliminated.

## 3. Discussion

The weighted-effect ASCA (WE-ASCA) was introduced as a new extension of the classical ASCA to analyze multivariate data in unbalanced multifactorial designs. The core of this WE-ASCA is to use the weighted-effect (WE) coding in designing model matrices of GLMs instead of dummy coding and deviation coding considered in designing schemes of classical ASCA and its extension ASCA+, respectively. The main advantage of implementing the WE-coding is that the sum of all level effects of a categorical variable in the design matrix is equal to zero for unbalanced designs, which is not the case by the other coding schemes. Also, the WE-coding offers a unique estimation of the effect of a specific variable because it always codes a variable with α levels by α−1 columns within the design matrix. The described advantages convinced us to update the coding scheme utilized in the design matrix of ASCA+ with the WE-coding. The response matrix then can be estimated easily by a general linear model and using the new balanced design matrix and the parameter matrix. This estimated response can be decomposed linearly as different effect matrices representing the experimental factors and their interactions. Besides, the significant effects in a particular design are determined based on permutation tests while the dimensions of the effect matrices are reduced using PCA.

Using a Raman spectral data set consisting of four colorectal tissue types that were collected from 47 mice in 387 scans, two possible applications of WE-ASCA were checked. Here, the data set was acquired with respect to four factors describing the experimental design. These factors are the different individuals with 47 levels referring to the mice, the activity of the P53 gene, the mouse gender, and the location of samples (colon or rectum). In the first application, we aimed to understand and analyze the design of our experiment in addition to determining which of experimental factors contributed significantly to the considered experiment. This was achieved by applying ASCA, ASCA+, and WE-ASCA and comparing their results based on the explained variances by all effects. It tuned out that the classical ASCA overestimated the effect contributions, while the ASCA+ underestimated these contributions. In contrast, the presented WE-ASCA performed the best in estimating these effect contributions in term of the summation of percentage of explained variances. Nevertheless, the three versions of ASCA proved that the individual factor has the largest effect in our design. Therefore, we studied the influence of excluding such variations on the classification of colorectal tissues. This was demonstrated for four different classification tasks using two classifiers, i.e., PCA-LDA and PLS-DA, and leave-one-mouse-out cross-validation as a validation method. Our results showed that excluding the contribution of the individual factor from the training set introduced more robust classification results, and it improved the mean sensitivity in most classification tasks. Additionally, the training of an LDA model on spectra, when their individual effects were excluded, required a smaller number of principal components (or latent variables) and improved the reproducibility of the results.

## 4. Methods

### 4.1. ASCA for Crossed Balanced Design

The typical way of depicting the ASCA starts by introducing the ANOVA decomposition for the response of a single measurement described by a single variable [23]. Therein, the variation of each cell in a response matrix $X\in {\mathbb{R}}^{n\times m}$, which has n measurements described by m variables (features), can be defined for a two-factor crossed design as:

(3) $$x_{ijpq}=\mu_{j}+a_{jp}+b_{jq}+ab_{jpq}+\varepsilon_{ijpq},\;i=1,\;2,\;\ldots ,\;n;j=1,\;2,\;\ldots ,\;m;p=1,\;2,\;\ldots ,\;\alpha ;\;q=1,\;2,\;\ldots ,\beta ,$$

where $x_{ijpq}$ is the observed response of a measurement, i, on the variable, j, that has the level p of factor A and the level q of factor B. The parameter $\mu_{j}$ indicates the global mean with respect to the variable j, and $\varepsilon_{ijpq}$ refers to the error term which is supposed to be a Gaussian distributed random variable with a mean of 0. Under additional constrains of ANOVA described in [13,17,23], a unique estimation of the previous statistical model is:

(4) $$x_{ijpq}=x_{.j..}+(x_{.jp.}-x_{.j..})+(x_{.j.q}-x_{.j..})+\left(x_{.jpq}-x_{.jp.}-x_{.j.q}+x_{.j..}\right)\;+\left(x_{ijpq}-x_{.jpq}\right).$$

The dot-notation in the previous equation’s subscripts describes over which index the mean is calculated. Moving to multivariate data, the classical ASCA provides a direct generalization of the ANOVA tests in balanced designs. It calculates the effect contributions to the response matrix X:

(5) $$X=M_{0}+Con_{A}+Con_{B}+Con_{AB}+\hat{E},$$

where $M_{0}\in {\mathbb{R}}^{n\times m}$ represents the global mean matrix (its rows are the means over the variables), $Con_{f}\in {\mathbb{R}}^{n\times m}$ refers to the estimated effect contribution of f where f∈{A,B,AB}, and $\hat{E}\in {\mathbb{R}}^{n\times m}$ estimates the residual matrix. When the experimental design is balanced, each estimated effect matrix in Equation (5) is orthogonal, and the summation of percentage of variances of these matrices equals to 100%. Consequently, the contributions of individual effects in the overall variance can be measured by the partitions of the following sums of squares:

(6) $$∥X∥{}^{2}=∥M_{0}∥{}^{2}+∥Con_{A}∥{}^{2}+∥Con_{B}∥{}^{2}+∥Con_{AB}∥{}^{2}+∥\hat{E}∥{}^{2},$$

where $∥.∥{}^{2}$ indicates the squared Frobenius norm.

### 4.2. General Linear Models and ASCA+

The general linear models (GLMs) usually refer to a multiple linear regression where a continuous response variable is given continuous and (or) categorical predictors. These GLMs are fundamentals for several statistical tests such as ANOVA and ANCOVA (analysis of covariance) [24]. In its multivariate version, GLMs aim to decompose a response matrix $X\in {\mathbb{R}}^{n\times m}$ linearly into different contributions based on a design matrix $D\in {\mathbb{R}}^{n\times p}$ and a parameter matrix $\beta \in {\mathbb{R}}^{p\times m}$. These contributions are related to the experimental factors and their interactions while the linear decomposition can be formulated as the following equation:

(7) X=Dβ+E,

where $E\in {\mathbb{R}}^{n\times m}$ denotes to the residual matrix. In formula (7), the matrix β relies only on the data features like intensity or pixel values while the method of coding the design (model) matrix D performs a critical part in GLMs decomposition. In principle, the matrix D can be designed using different coding techniques; however, a unique estimation of effect contributions can be obtained only if a factor with α levels is coded by α−1 columns in the design matrix [17]. By ASCA+, the deviation coding was utilized to design the matrix D, where a factor with α levels is coded by α−1 columns with values of 0 and 1 for the first α−1 levels and with the value of −1 for the last level of this factor [17]. Then the parameter matrix β is estimated using the ordinary least square method as $\hat{\beta }={\left(D^{T}D\right)}^{-1}D^{T}X$, and the data matrix X is approximated as $\hat{X}=D\;\hat{\beta }$. The error of this estimation is determined by the residual matrix $\hat{E}$ which can be written mathematically as:

(8) $$\hat{E}=X-D\hat{\beta }.$$

Nevertheless, the main advantage of the previous coding is that the sub-design matrix of each effect is orthogonal for a balanced design. For instance, suppose a balanced two-factor crossed design is considered in which six measurements are affected by the factors $A:\left(a_{1},\;a_{2}\right)$ and $B:\;(b_{1},\;b_{2},\;b_{3})$. A response matrix $X\in {\mathbb{R}}^{6\times m}$ of this balanced design can be described with respect to these factor levels as:

(9) $$X={\left[X_{1,a_{1}b_{1}}\;X_{2,a_{1}b_{2}}\;X_{3,a_{1}b_{3}}\;X_{4,a_{2}b_{1}}\;X_{5,a_{2}b_{2}}\;X_{6,a_{2}b_{3}}\right]}^{T}.$$

$X_{i,a_{k}b_{l}}\in {\mathbb{R}}^{m}$ indicates a measurement of level $a_{p}:p\in \left\{1,\;2\right\}$ and level $b_{q}:q\in \left\{1,\;2,\;3\right\}$, which is oriented in the row i∈{1, 2,…, 6}. Based on the ASCA+ and the deviation coding, the design matrix D and the sub-design matrix of factor B can be visualized by:

$$D=\left[\begin{array}{cccccc} M_{0} & A & b_{1} & b_{2} & A:b_{1} & A:b_{2}\\ 1 & 1 & 1 & 0 & 1 & 0\\ 1 & 1 & 0 & 1 & 0 & 1\\ 1 & 1 & -1 & -1 & -1 & -1\\ 1 & -1 & 1 & 0 & -1 & 0\\ 1 & -1 & 0 & 1 & 0 & -1\\ 1 & -1 & -1 & -1 & 1 & 1 \end{array}\right];D_{/B}=\left[\begin{array}{cccccc} M_{0} & A & b_{1} & b_{2} & A:b_{1} & A:b_{2}\\ 0 & 0 & 1 & 0 & 0 & 0\\ 0 & 0 & 0 & 1 & 0 & 0\\ 0 & 0 & -1 & -1 & 0 & 0\\ 0 & 0 & 1 & 0 & 0 & 0\\ 0 & 0 & 0 & 1 & 0 & 0\\ 0 & 0 & -1 & -1 & 0 & 0 \end{array}\right].$$

Then, the effect matrix of factor B, namely $Con_{B}$, is simply estimated by multiplying the matrix $D_{/B}$ and the parameter matrix $\hat{\beta }$. Likewise, all sub-design matrices and effect matrices can be calculated, and the response matrix X is subsequently decomposed according to formula (3) while the contribution of each effect into overall variance is estimated using a squared Frobenius norm (see Equation (6)).

### 4.3. Weighted-Effect ASCA (WE-ASCA)

The deviation coding introduced by ASCA+ provides an orthogonal design matrix only if an experimental design is balanced. In this case, the traditional constrains of analysis of variance models are perfectly satisfied, and the summation of percentage of variance equals to 100%. For unbalanced multifactorial designs, the design matrices introduced by any of ASCA or ASCA+ are non-orthogonal, and the estimated experiment effects are biased; therefore, it is desirable to remove, or at least reduce, these estimation biases. Another coding scheme, which can be considered to design the model matrix of GLMs in unbalanced data, is the weighted-effect (WE) coding. This WE-coding is a type of dummy coding which can be used to facilitate the inclusion of categorical variables in GLMs [19,20]. Thereby, the effect of each level of a categorical variable represents the level deviation from the weighted mean instead of using the grand mean in deviation coding. The WE-coding offers an attractive property related to the constrain in which the sum of all level effects of a categorical variable is equal to zero, which is not fulfilled by other coding schemes in unbalanced designs [19]. Moreover, the results of WE-coding are identical with those obtained by deviation coding if an experiment’s design is balanced. Beside this, the estimated effect of a specific variable provided by the WE-coding are unique because a variable of α levels is coded by α−1 columns within the model matrix, and the interaction between two variables of α and β levels is coded by (α−1)×(β−1) columns in this model matrix. Using the previous advantages, the WE-coding can be used to improve the performance of ASCA models in unbalanced multifactorial designs. In the following, the implementation of WE-coding in unbalanced multifactorial designs will be described in detail for a two-factor crossed design. However, this coding scheme is still valid for higher multifactorial designs. Let $X\in {\mathbb{R}}^{7\times m}$ be a response matrix collected from an unbalanced two-factor crossed design and presented as:

(10) $$X={\left[X_{1,a_{1}b_{1}}\;X_{2,a_{1}b_{2}}\;X_{3,a_{1}b_{3}}\;X_{4,a_{2}b_{1}}\;X_{5,a_{2}b_{2}}\;X_{6,a_{2}b_{3}}\;X_{7,a_{2}b_{3}}\right]}^{T},$$

where $X_{i,a_{k}b_{l}}\in {\mathbb{R}}^{m}$ indicates a measurement of level $a_{p}:p\in \left\{1,\;2\right\}$ and level $b_{q}:q\in \left\{1,\;2,\;3\right\}$, which is oriented in rows i∈{1, 2,…, 7}. According to ASCA+, the design matrix D and the sub-design matrix with respect to factor B can be depicted by:

$$D=\left[\begin{array}{cccccc} M_{0} & A & b_{1} & b_{2} & A:b_{1} & A:b_{2}\\ 1 & 1 & 1 & 0 & 1 & 0\\ 1 & 1 & 0 & 1 & 0 & 1\\ 1 & 1 & -1 & -1 & -1 & -1\\ 1 & -1 & 1 & 0 & -1 & 0\\ 1 & -1 & 0 & 1 & 0 & -1\\ 1 & -1 & -1 & -1 & 1 & 1\\ 1 & -1 & -1 & -1 & 1 & 1 \end{array}\right];D_{/B}=\left[\begin{array}{cccccc} M_{0} & A & b_{1} & b_{2} & A:b_{1} & A:b_{2}\\ 0 & 0 & 1 & 0 & 0 & 0\\ 0 & 0 & 0 & 1 & 0 & 0\\ 0 & 0 & -1 & -1 & 0 & 0\\ 0 & 0 & 1 & 0 & 0 & 0\\ 0 & 0 & 0 & 1 & 0 & 0\\ 0 & 0 & -1 & -1 & 0 & 0\\ 0 & 0 & -1 & -1 & 0 & 0 \end{array}\right].$$

We can note that the design matrix D is non-orthogonal and that the sum of level effects of the factors and their interaction does not equal to zero, which introduces biased estimators of experimental effects. The previous design matrix can be converted into a balanced design matrix if the WE-coding described by the coding matrix in Table 4 is applied. Based on this table, the obtained balanced design matrix BD and the balanced sub-design matrix $BD_{/B}$ are determined as the following:

$$BD=\left[\begin{array}{cccccc} M_{0} & A & b_{1} & b_{2} & A:b_{1} & A:b_{2}\\ 1 & 1 & 1 & 0 & 1 & 0\\ 1 & 1 & 0 & 1 & 0 & 1\\ 1 & 1 & -2/3 & -2/3 & -1/1 & -1/1\\ 1 & -3/4 & 1 & 0 & -1/1 & 0\\ 1 & -3/4 & 0 & 1 & 0 & -1/1\\ 1 & -3/4 & -2/3 & -2/3 & 1/2 & 1/2\\ 1 & -3/4 & -2/3 & -2/3 & 1/2 & 1/2 \end{array}\right];$$

$$BD_{/B}=\left[\begin{array}{cccccc} M_{0} & A & b_{1} & b_{2} & A:b_{1} & A:b_{2}\\ 0 & 0 & 1 & 0 & 0 & 0\\ 0 & 0 & 0 & 1 & 0 & 0\\ 0 & 0 & -2/3 & -2/3 & 0 & 0\\ 0 & 0 & 1 & 0 & 0 & 0\\ 0 & 0 & 0 & 1 & 0 & 0\\ 0 & 0 & -2/3 & -2/3 & 0 & 0\\ 0 & 0 & -2/3 & -2/3 & 0 & 0 \end{array}\right].$$

Here, each effect A, B, and AB is estimated in BD by p−1=1, q−1=2, and (p−1)×(q−1)=2 columns, respectively. Clearly, the considered WE-coding estimates the levels of effects A, B, and AB in a way that the sum is always equal to zero.

In this paper, we update the ASCA+ by replacing the deviation coding of the design matrix in GLMs by the WE-coding. This updated version, namely weighted-effect ASCA (WE-ASCA), provides a new extension of ASCA in unbalanced multifactorial designs. Thereby, a balanced design matrix BD is estimated using the WE-coding [19,20], then the parameter matrix β is estimated based on the ordinary least square method:

(11) $$\hat{\beta }={\left(BD^{T}BD\right)}^{-1}\;BD^{T}\;X.$$

The response matrix X can be thereafter approximated as $\hat{X}=BD\;\hat{\beta }$, while the error of this estimation is given by:

(12) $$\hat{E}=X-BD\;\hat{\beta }.$$

Because the WE-coding estimates each factor of α levels by α−1 columns in the design matrix BD, the presented WE-ASCA analysis provides a unique solution to solve the statistical models of any multifactorial design. Additionally, it reduces the bias introduced by unbalanced designs. Consequently, the effect matrix of any factor or interaction in multifactorial designs can be uniquely estimated by:

(13) $$Con_{f}=BD_{/f}\;\hat{\beta },$$

where $BD_{/f}$ and $Con_{f}$ denotes the balanced sub-design matrix and the effect matrix of factor (or interaction) f, respectively. In case of a two-factor crossed design, the response matrix X is decomposed linearly into different effects of the factors A and B and their interaction:

(14) $$X=M_{0}+Con_{A}+Con_{B}+Con_{AB}+\hat{E}.$$

The previous estimated effect matrices have the same size of matrix X. Thus, it is useful to reduce the dimensions of these matrices using PCA models in order to highlight the variations between different effect levels. In the presented two-factor crossed design, the statistical model based on the PCA sub-models can be presented by the decomposition:

(15) $$X=M_{0}+T_{A}P_{A}^{T}+T_{B}P_{B}^{T}+T_{AB}P_{AB}^{T}+\hat{E},$$

where the matrix $T_{f}$ represents the score matrix, which highlights the variations between the levels of effect f. The matrix $P_{f}$ denotes the loadings matrix of the same effect.

### 4.4. The Percentage of Variance

One of the main goals of multifactorial design analysis is to study how different factors influence a particular experiment based on estimating their contributions to the overall variance. In balanced designs, a factor contribution is approximated simply using the type I sum squares. This method of sum squares sequentially computes the factor contributions with respect to their order in the designed model [25]. For the unbalanced multifactorial design, the factor levels have different numbers of measurements, which provides overestimation of some factor contributions. It is recommended according to [8,25,26] to calculate these contributions based on the type III sum squares. Thereby, the effect of one factor is evaluated after all other factors have been considered. This type of sum squares offers identical estimations of factor contributions with those obtained by type I sum squares when the considered design is balanced. In Table 5, the type III sum squares of each effect contribution in a two-factor crossed design is presented. Herein, the mean model decomposes the response matrix X based on the global mean matrix $M_{0}$ and the overall variance, named ${\hat{E}}_{1}$. Then, the response matrix X is decomposed by a reduced model that does not consider the contribution of a specific effect f. Subsequently, the residual matrix ${\hat{E}}_{f}$ of this reduced model is estimated, and the sum squares of the effect f is calculated by the difference between $∥{\hat{E}}_{f}∥{}^{2}$ and $∥\hat{E}∥{}^{2}$. The explained variance by each effect can be approximated finally as a percentage of the sum squares of that effect to the sum squares of the residual matrix of the mean model, i.e., $\mathrm{SS}\left({\hat{E}}_{1}\right)$. Mathematically, the percentage of variance explained by an effect f ∈{A, B, AB} and the percentage of variance explained by the residual $\hat{E}$ of a two-factor crossed model can be formulated as:

(16) $$\%Var_{f}=\frac{∥{\hat{E}}_{f}∥{}^{2}-∥\hat{E}∥{}^{2}}{∥X-M_{0}∥{}^{2}}\times 100\;\mathrm{and}\;\;\%Var_{\hat{E}}=\frac{∥\hat{E}∥{}^{2}}{∥X-M_{0}∥{}^{2}}\times 100.$$

In our study, we compare the results of the WE-ASCA with the classical ASCA and its extension ASCA+ based on the summation of percentage of variances in an unbalanced design.

### 4.5. Permutation Tests

The basic idea of permutation tests is to check whether a specific effect f in ANOVA models contributes significantly to the variation of an experiment or it has a random influence [14,17]. For a two-factor crossed design, the procedure of permutation test starts by calculating the Frobenius sum squares of the first l principal components of the score matrix $T_{f}$ for each effect f∈{A, B, AB}:

(17) $$SS\left(f\right)=\overset{n}{\underset{i=1}{\sum }}\overset{l}{\underset{j=1}{\sum }}{\left(T_{f}\right)}_{i,j}^{2},$$

where n denotes the number of measurements in a response matrix $X\in {\mathbb{R}}^{n\times m}$. Then, we generate N random permutations of the rows of X, and the Frobenius norm $SS_{r}\left(f\right)$ of each effect is computed for each permutation r=1, 2,…,N with respect to the considered ASCA extension, i.e., ASCA+ and WE-ASCA. The last step of this test is to calculate the p-value p(f) as:

(18) $$p\left(f\right)=\frac{\#\left\{SS_{r}\left(f\right)\geq SS\left(f\right)\right\}}{N}.$$

This p-value determines whether an effect, *f*, explains a random variation or shows a significant contribution within a considered experiment.

### 4.6. Data and Software

All computational parts were carried out based on in-house written functions in R version 3.4.2. The utilized Raman spectral data and R functions are freely available via Zenodo through the following links:

- Raman spectra of colon cancer in a mice model: https://zenodo.org/deposit/3975464
- Weighted-effect ASCA (WE-ASCA) codes: https://zenodo.org/deposit/3975471

## 5. Conclusions

The presented WE-ASCA provides an updated version of the ASCA and ASCA+ that suits the analysis of variance in unbalanced multifactorial designs. WE-ASCA proved its potential in understanding and analyzing the influence of experimental factors in a complex multifactorial design. Furthermore, the WE-ASCA was presented as a powerful preprocessing tool that can improve the classification performance and increase the classification reproducibility. The current implementations of WE-ASCA were checked only for Raman spectra and for tissue classification tasks; however, the application field is not limited to these previous applications. It can be extended to cover the analysis of variance of any type of multivariate data and any statistical modeling task.

## Figures and Tables

**Table 1:** The variance explained by the experimental effects in percentage (%) using ANOVA-simultaneous component analysis (ASCA), ASCA+ and WE-ASCA. A value of zero represents a percentage smaller than $10^{-9}$.

Effect	Individual	Location	P53 Gene	Gender	Location: P53	Location: Gender	P53: Gender	Residuals	Sum (%)
ASCA	37.96	1.47	1.07	1.03	0.25	0.28	0.94	65.06	108.62
ASCA+	33.00	0.53	0	0	0.19	0.18	0	59.38	93.3
WE-ASCA	33.94	0.61	0	0	0.20	0.19	0	61.07	96.01

**Table 2:** The percentage (%) of variance explained by the first two principal components of the PCA models. The mice data set and effect matrices obtained by ASCA, ASCA+, and WE-ASCA are fitted with a PCA model.

Effect		All Data	Individuals	Location	Location: P53	Location: Gender
ASCA	PC1 (%)	42.52	36.44	100	97.16	97.10
	PC2 (%)	15.20	17.74	0	2.20	1.7
ASCA+	PC1 (%)	42.52	38.78	100	100	100
	PC2 (%)	15.20	17.40	0	0	0
WE-ASCA	PC1 (%)	42.52	35.49	100	100	100
	PC2 (%)	15.20	18.19	0	0	0

**Table 3:** The obtained *p*-values p(f) based on applying the permutation test on the results of ASCA+ and WE-ASCA analyses.

Effect		Individuals	Location	Location: P53	Location: Gender
p(f)	ASCA+	0.000	0.034	0.399	0.453
	WE-ASCA	0.000	0.021	0.383	0.424

**Table 4:** The coding matrix of a two-factor crossed design based on the weighted-effect coding. In this design, the effects of factors $A:\left(a_{1},\;a_{2}\right)$, $B:\left(b_{1},\;b_{2},\;b_{3}\right)$ and their interaction AB are presented.

Possible Combinations	Factors			Interaction	
	A	$b_{1}$	$b_{2}$	$A:b_{1}$	$A:b_{2}$
$a_{1}\&b_{1}$	1	1	0	1	0
$a_{1}\&b_{2}$	1	0	1	0	1
$a_{1}\&b_{3}$	1	$-n_{b_{1}}/n_{b_{3}}$	$-n_{b_{2}}/n_{b_{3}}$	$-n_{a_{1},\;b_{1}}/n_{a_{1},\;b_{3}}$	$-n_{a_{1},\;b_{2}}/n_{a_{1},\;b_{3}}$
$a_{2}\&b_{1}$	$-n_{a_{1}}/n_{a_{2}}$	1	0	$-n_{a_{1},\;b_{1}}/n_{a_{2},\;b_{1}}$	0
$a_{2}\&b_{2}$	$-n_{a_{1}}/n_{a_{2}}$	0	1	0	$-n_{a_{1},\;b_{2}}/n_{a_{2},\;b_{2}}$
$a_{2}\&b_{3}$	$-n_{a_{1}}/n_{a_{2}}$	$-n_{b_{1}}/n_{b_{3}}$	$-n_{b_{2}}/n_{b_{3}}$	$n_{a_{1},\;b_{1}}/n_{a_{2},\;b_{3}}$	$n_{a_{1},\;b_{2}}/n_{a_{2},\;b_{3}}$

**Table 5:** Type III sum squares for a two-factor crossed design.

Model		Type III Sum Squares
Mean model	$X=M_{0}+{\hat{E}}_{1}$	$SS\left({\hat{E}}_{1}\right)=∥X-M_{0}∥{}^{2}$
Two-factor cross design	$X=M_{0}+Con_{A}+Con_{B}+Con_{AB}+\hat{E}$	$SS\left(\hat{E}\right)=∥\hat{E}∥{}^{2}$
Without the effect of A	$X=M_{0}+Con_{B}+Con_{AB}+{\hat{E}}_{A}$	$SS\left(Con_{A}\right)=∥{\hat{E}}_{A}∥{}^{2}-∥\hat{E}∥{}^{2}$
Without the effect of B	$X=M_{0}+Con_{A}+Con_{AB}+{\hat{E}}_{B}$	$SS\left(Con_{B}\right)=∥{\hat{E}}_{B}∥{}^{2}-∥\hat{E}∥{}^{2}$
Without the effect of AB	$X=M_{0}+Con_{A}+Con_{B}+{\hat{E}}_{AB}$	$\left(Con_{AB}\right)=∥{\hat{E}}_{AB}∥{}^{2}-∥\hat{E}∥{}^{2}$

## Data Availability

The data of Vogler et al. [21] and scripts are available in the following repositories: Raman spectra of colon cancer in a mice model: https://zenodo.org/deposit/3975464; Weighted-effect ASCA (WE-ASCA) codes: https://zenodo.org/deposit/3975471.
